# Strangulated intercostal liver herniation subsequent to blunt trauma. First report with review of the world literature

**DOI:** 10.1186/1749-7922-7-23

**Published:** 2012-07-16

**Authors:** Cino Bendinelli, Andrew Martin, Shane D Nebauer, Zsolt J Balogh

**Affiliations:** 1 Department of Traumatology, John Hunter Hospital, Newcastle, NSW, Australia; 2 University of Newcastle, Newcastle, NSW, Australia

**Keywords:** Strangulated, Intercostal hernia, Liver herniation, Blunt trauma, Laparoscopic mesh repair

## Abstract

Traumatic transdiaphragmatic intercostal hernia, defined as an acquired herniation of abdominal contents through disrupted intercostal muscles, is a rarely reported entity. We present the first reported case of a traumatic transdiaphragmatic intercostal hernia complicated by strangulation of the herniated visceral contents.

Following blunt trauma, a 61-year old man developed a traumatic transdiaphragmatic intercostal hernia complicated by strangulation of liver segment VI. Due to pre-existing respiratory problems and the presence of multiple other injuries (grade III kidney laceration and lung contusion) the hernia was managed non-operatively for the first 2 weeks.

The strangulated liver segment eventually underwent ischemic necrosis. Six weeks later the resulting subcutaneous abscess required surgical drainage. Nine months post injury the large symptomatic intercostal hernia was treated with laparoscopic mesh repair. Twelve months after the initial trauma, a small recurrence of the hernia required laparoscopic re-fixation of the mesh.

This paper outlines important steps in managing a rare post traumatic entity. Early liver reduction and hernia repair would have been ideal. The adopted conservative approach caused liver necrosis and required staged procedures to achieve a good outcome.

## Introduction

Traumatic transdiaphragmatic intercostal hernia (TTIH) is a rare pathology with only sporadic cases published in the literature [1-21]. TTIH is defined as an acquired herniation of the abdominal contents through intercostal muscles [1-21]. The condition generally occurs following the disruption of intercostal muscles and the diaphragm as a consequence of either blunt [1-13] or penetrating trauma [5,13-15]. However, in elderly and demented patients TTIH following strenuous coughing have been reported [16-18]. To date, there are no published cases describing a TTIH complicated by strangulation of the herniated visceral contents. We report the case of a TTIH with associated strangulation and necrosis of segment VI of the liver.

Statement of approval by Local Ethical Committee and patient was obtained.

## Case report

### Stage 1. Acute

A 61-year old man was admitted at Level 1 Trauma Centre, following a 3 metre fall from scaffolding onto a trestle stand. On arrival the patient showed normal vital signs and was complaining of pain in the right thoracoabdominal region, where a seriously injured skin mark and swelling was obvious. A right haemopneumothorax was identified on chest X-ray and treated with a 32Fr chest tube. Computer tomography (CT) with intravenous contrast demonstrated: right lung contusions, lateral 9^th^ to 12^th^ rib fractures with herniation of segment VI of the liver through an acquired defect in the 9^th^ -10^th^ intercostal space, a grade III liver laceration and a grade III laceration of right kidney without contrast extravasation. Medical history included: obesity, hypertension, and obstructive sleep apnoea requiring a continuous positive airway pressure device at night.

**Figure 1:**
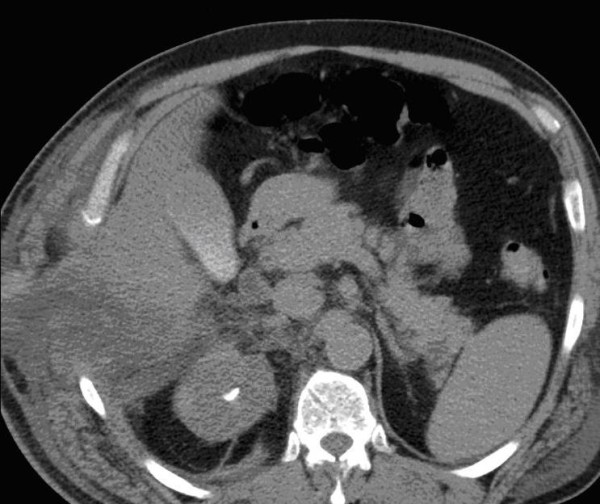
CT at 48 hours post injury: herniated segment VI of the liver without contrast enhancement, suggesting strangulation.

The initial management of these injuries was conservative. The patient required High Dependency Unit admission for non invasive ventilation, pain relief and aggressive chest physiotherapy. Follow-up CT (48 hours postinjury) demonstrated the absence of contrast enhancement suggesting strangulation of the herniated liver (Figure 1). Transaminases and all liver function test were only slightly elevated. Conservative management was successful and the patient was discharged 12 days post injury.

### Stage 2. Sub Acute

**Figure 2:**
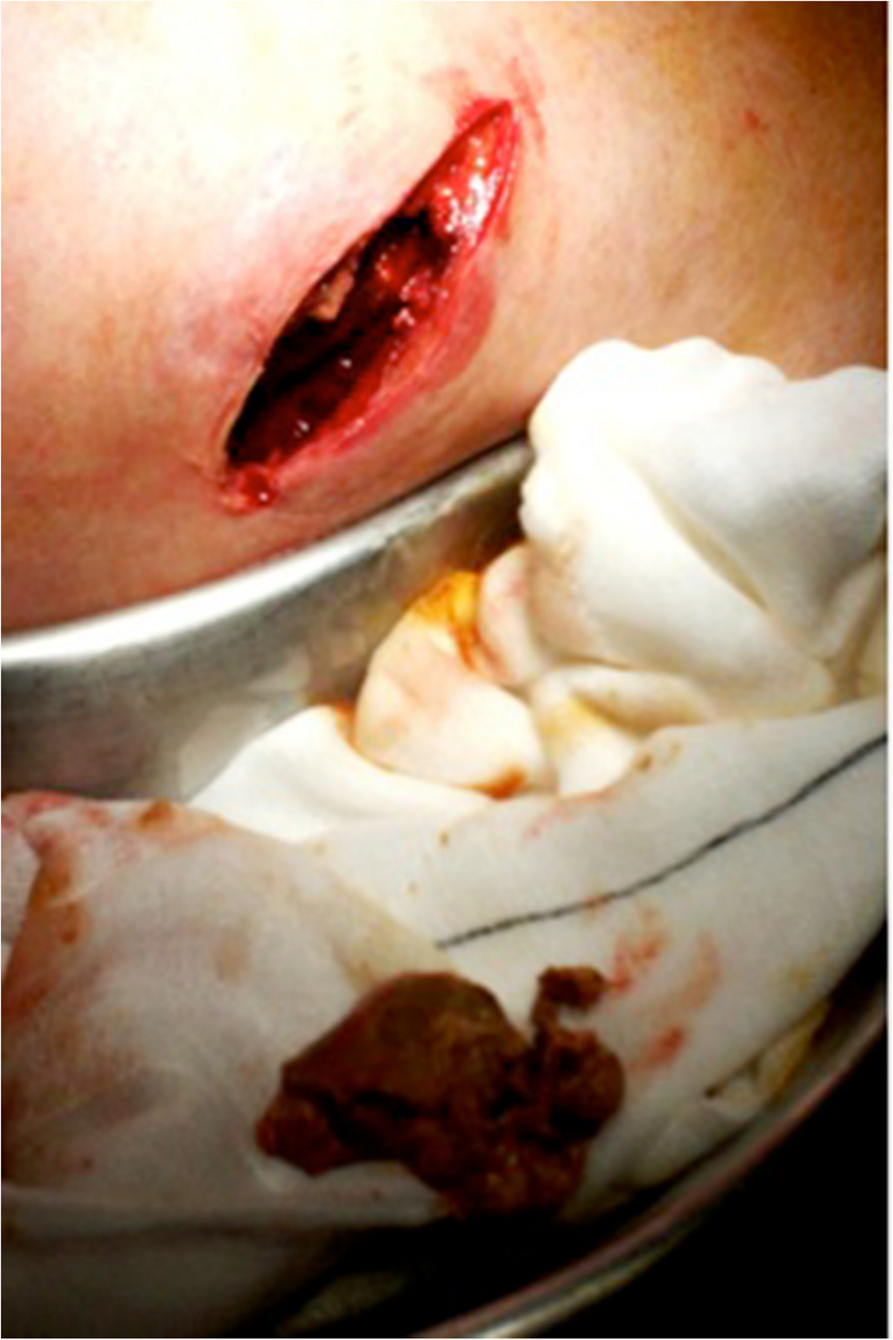
Incision and drainage of subcutaneous collection containing necrotic liver.

At 45 days follow-up the patient presented with a large and painful collection (70 x 65 mm). This was treated with incision and drainage. About 50 ml of necrotic liver was debrided (Figure 2). Definitive repair of the TTIH was further postponed due to the risk of a prosthetic mesh infection. Intra-operative cultures taken however showed no growth.

### Stage 3. Chronic

**Figure 3:**
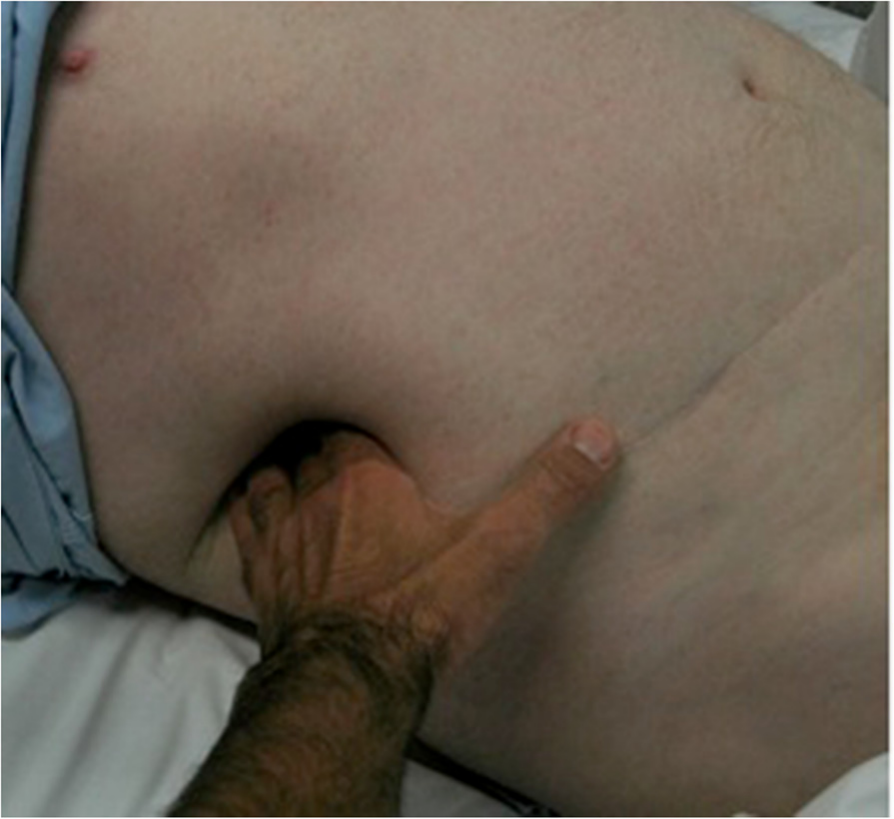
Easily reducible TTIH.

**Figure 4:**
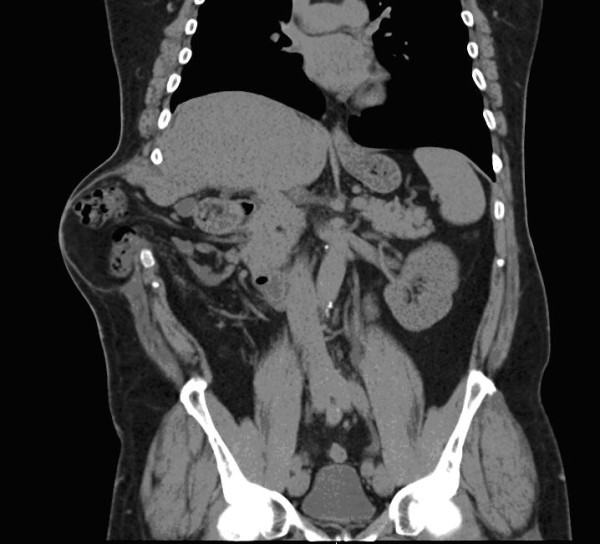
Coronal CT view: Hepatic colonic flexure and some liver tissue are included in the sac of TTIH.

At 7 months follow-up, the patient presented with a large reducible TTIH (Figure 3). On CT, the defect measured 120 x 90 mm and the sac contained the hepatic flexure of the colon and a small part of the liver margin (Figure 4). The repair of the defect was planned in 2 months in order to allow full recovery from injury and optimization of body weight.

Definitive surgical repair was performed under general anaesthetic, with the patient on left lateral decubitus position. Laparoscopic port placement involved a 10 mm umbilical port, one 15 mm port and two 5 mm ports in equidistant subcostal positions. After initial orientation, the hepatic flexure, the omentum and the liver margin were sharply dissected from the sac. Once the sac and its neck were clearly demonstrated, a 21.0x15.9 cm low profile polypropylene and expanded polytetrafluoroethylene (ePTFE) double mesh prosthesis (Bard® Composix® L/P Mesh, US) was used for the repair. Due to the proximity of the diaphragm to the defect, it was decided to use a combination of intracorporal suturing and endoscopic tacks. The caudal part of the mesh was secured to the abdominal wall with helical tacks (5 mm Protack® Autosuture® Tyco®, US). The cranial aspect of the mesh was sutured to the diaphragm with a continuous 1 braided polyester (CT-1 Ethibond®, US). The postoperative course was uneventful, with hospital discharge on the fourth postoperative day.

At the twelve months follow up after hernia repair the patient presented with some discomfort and features suggesting a recurrent hernia. CT confirmed the diagnosis and identified the presence of omentum in the sac.

At laparoscopic exploration the mesh appeared well embodied and completely peritonealised. There was a 2 x 2 cm defect between the abdominal wall and the lower part of the mesh (due to failure of the endotack fixation). The omentum was reduced in the abdomen and the mesh sutured to abdominal wall by laparoscopic means. In order to increase the strength of the repair, the intercostal space was partially re-approximated with three figure of eight braided polyester stiches (CT-1 Ethibond®, US) through a 5 cm incision. The patient was discharged 48 hours post procedure with minimal discomfort. At the 12-month follow up after the second reconstructive procedure there was no evidence of recurrence.

## Discussion

TTIH is rare sequelae of injury. In 1911 Gerster already challenged this concept. He reviewed 10 cases and concluded “that the occurrence of these herniae is not as rare as the few published communications on this subject would lead one to believe” [13]. TTIH are most commonly the result of penetrating injuries [5,13-15] or high energy and focused blunt strikes [1-13]. More frequently seen on the left side, TTIH may contain omentum, colon, spleen, stomach, and/or small bowel. The diagnosis of TTIH has historically been difficult to make, with delayed diagnosis to up to several years [5,13]. On initial clinical examination, intercostal hernias have been mistaken for lipomas or hematomas [3]. In these cases, it was not until a CT that the diagnosis of intercostal herniation was confirmed.

We know of no reports in the literature in which a TTIH was associated with liver strangulation. The closest, albeit clearly different, reported cases being a left TTIH due to coughing with infarcted omentum found at elective repair [16] and a patient with Chilaiditi’s syndrome who required ileocecal resection during repair of a non-traumatic intercostal incisional hernia [22]. Conservative management of TTIH has been reported. Most often the patient presents with pain and increasing lump size and the repair is then considered [4].

The decision to elect the non-interventional approach despite liver strangulation was dictated by the patient’s comorbidities, severe lung contusion, non-operatively managed abdominal solid organ injuries (kidney, liver), partial thickness skin necrosis and the lack of compromised liver function. More aggressive operative approach could have prevented later readmissions but also could have resulted in severe complications such as major bleeding, respiratory failure and wound/mesh infection. This dilemma cannot be addressed by case studies of this rare injury, but our example highlights what can be expected with conservative approach. Whether this is applicable to a given patient to a given time requires the informed judgement of the treating surgeon.

Several repair techniques have been described: endogenous tissue repair [8], prosthetic mesh reinforced by cable banding around the ribs [18], open transthoracic mesh repair [20] and tension free laparoscopic absorbable mesh repair [21]. We favoured the laparoscopic tension-free approach and the use of a non absorbable dual layer mesh. The choice of a running suture for mesh fixation to the diaphragm was based upon manufacturer warnings, which contraindicate helical tacks for use in tissues less than 4 mm thick. The thickness of the diaphragm has been measured by ultrasound as low as 2 mm [23]. As a matter of fact, a fatal injury of the heart has been reported during hiatus hernia repair with helical tack [24].

## Conclusion

TTIH are rarely encountered and may be difficult to diagnose and treat without relevant imaging and preoperative planning. Liver strangulation, if not treated promptly, results in liver necrosis and mandates a staged surgical management of TTIH. Laparoscopic tension-free repair with a permanent prosthetic mesh and the use of suture for fixation to diaphragm are keys for a successful outcome.

## Competing interests

The authors declare that they have no competing interests.

## Authors’ contributions

CB and AM performed the surgical procedures and wrote the paper. SDN helped in data collection and in writing the paper. ZJB provided critical analysis and reviewed the paper. All authors read and approved the final manuscript.
